# Preparing for the data revolution: identifying minimum health information competencies among the health workforce

**DOI:** 10.1186/s12960-015-0002-x

**Published:** 2015-04-01

**Authors:** Maxine Whittaker, Nicola Hodge, Renata E Mares, Anna Rodney

**Affiliations:** School of Public Health, The University of Queensland, Herston Road, Brisbane, Australia

**Keywords:** Human resources for health, HRH, Health information system, HIS, Health systems, Competency, Low- to middle-income countries, LMICs, Competency frameworks

## Abstract

**Background:**

Health information is required for a variety of purposes at all levels of a health system, and a workforce skilled in collecting, analysing, presenting, and disseminating such information is essential to fulfil these demands. While it is established that low- and middle-income countries (LMICs) are facing shortages in human resources for health (HRH), there has been little systematic attention focussed on non-clinical competencies. In response, we developed a framework that defines the minimum health information competencies required by health workers at various levels of a health system.

**Methods:**

Using the Delphi method, we consulted with leading global health information system (HIS) experts. An initial list of competencies and draft framework were developed based on results of a systematic literature review. During the second half of 2012, we sampled 38 experts with broad-based HIS knowledge and extensive development experience. Two rounds of consultation were carried out with the same group to establish validity of the framework and gain feedback on the draft competencies. Responses from consultations were analysed using Qualtrics® software and content analysis.

**Results:**

In round one, 17 experts agreed to participate in the consultation and 11 (65%) completed the survey. In the second round, 11 experts agreed to participate and eight (73%) completed the survey. Overall, respondents agreed that there is a need for all health workers to have basic HIS competencies and that the concept of a minimum HIS competency framework is valid. Consensus was reached around the inclusion of 68 competencies across four levels of a health system.

**Conclusions:**

This consultation is one of the first to identify the HIS competencies required among general health workers, as opposed to specialist HIS roles. It is also one of the first attempts to develop a framework on minimum HIS competencies needed in LMICs, highlighting the skills needed at each level of the system, and identifying potential gaps in current training to allow a more systematic approach to HIS capacity-building.

**Electronic supplementary material:**

The online version of this article (doi:10.1186/s12960-015-0002-x) contains supplementary material, which is available to authorized users.

## Background

### Health information systems and human resources

It has indeed become a “truism” to assert that sound and reliable information is the foundation of all aspects of health system decision-making and that health information systems (HIS) are a core component of any effective health system [1]. More recently, the post-2015 development agenda has called for a “data revolution” with new initiatives to improve the quality of statistics and information available [2]. Across all levels of a health system, health information is required for a variety of purposes: from enhancing patient care and measuring facility outcomes, management, and resource allocation, to strategic planning. A workforce skilled in collecting, analysing, interpreting, presenting, and disseminating health information is essential to fulfil these demands. The human resources—people who have the knowledge, skills, and expertise to make the system work efficiently and effectively—are a key component. In a recent publication, for example, the authors emphasised how the “revolution in health information and systems” requires a strong, adequately skilled workforce [3].

Numerous low- and middle-income countries (LMICs) are facing shortages in human resources for health (HRH), and this is having negative impacts on the performance of health systems [4,5]. This shortage in absolute numbers is further compounded by the unequal distribution of workers across countries and regions, skill-mix imbalance, and weak knowledge base [4,6,7]. While many initiatives have been directed at these HRH issues, they have mostly focused on the clinical HRH required for prevention, care, and treatment [8]. To date, there has been little systematic attention focused on the non-clinical competencies required among HRH, such as data collection and use [6,9,10].

While there are cadres of workers with information-specific roles, such as data-entry clerks, clinical coders, and statisticians, the reality is that information management is a central part of all health workers’ roles [11]. Previous research has estimated that physicians spend between 25% and 40% of their time on documentation and information management [11,12]. Nurses also spend a considerable amount of time completing monthly data summaries and updating facility and patient registers [9]. Information is needed to assist with a number of patient care functions, including administrative, legal, and financial, as well as helping to prevent, heal, and ease disease [11,13].

However, many health staff do not see HIS tasks as part of their role but more of an unwelcome burden that detracts from their primary role as service providers [14]. HIS tasks and competencies are often not well defined in position descriptions for healthcare workers, especially in LMICs where staff may be required to perform multiple roles simultaneously [7,15,16]. While it has been acknowledged that every health worker needs to have an “understanding of the value of health data… as a prerequisite for maintaining patient/client safety”, it appears that many have failed to appreciate this need [17], with few initiatives focussing on building HIS competencies among the health workforce.

### The need for competency frameworks

Competency-based frameworks are common among health professions in high-income countries and are frequently used to define standards for education and training and for career progression [18]. Research shows that competency modelling can identify relevant groupings of knowledge, skills, abilities, and other characteristics that affect an individual’s role or responsibilities; relate to job performance; are measurable; and are subject to improvement through training and development activities [19]. Having clearly defined and objectively verifiable competencies are also important in ensuring staff ability to perform specific tasks, for which there can be major discrepancies between perceived and actual competence [9,20,21].

While a considerable amount of research has focused on informatics competencies needed in public health, these have mostly focused on the biomedical [11-13,22] or nursing [23-25] professions. Several studies have described non-clinical competencies required among health staff; however, these have again focussed on informatics and are primarily based in high-income countries [17,26-30]. Overall, there is a noticeable paucity of evidence on the broad health information competencies that health workers need to effectively carry-out their roles, especially in LMICs.

### Consultation aims

The goal of the expert consultations was to create a framework that defines the minimum HIS competencies required by health workers at various levels of health systems in LMICs. Such a framework will inform the HIS skills and abilities that the workforce requires and enable the education and training sectors to align learning outcomes to these. This will assist individuals and organisations with career planning and allow the health sector to implement useful professional development. In addition, it will provide planners and policymakers with a level of certainty regarding the desired mix and quality of skill sets at various levels of the health system and influence strategies for designing the workforce.

Although many LMICs are embracing health informatics, particularly in Africa and Asia, many still use conventional paper-based systems for collecting health information and are unlikely to rely on advanced technologies. Paper-based systems require workers to have specific information competencies to deal with their shortcomings, especially in addressing issues such as transposing errors, repeated manual data entry, and a lack of advanced analytical tools. This consultation aims to identify the minimum HIS competencies that could be expected in LMICs that do not have advanced technology.

## Methods

The overall methodology was based on a literature review to inform a modified Delphi approach to agree on levels of practice within a health system and associated health information competencies with each level. We consulted with leading global health information experts about the minimum HIS competencies health staff (who are not involved in specialist HIS operations) need at various levels of the health system in LMICs. To provide the necessary framework and guide interviews, we adopted the following working definition of “competency” as follows: the knowledge, skills, abilities, and/or qualifications required to adequately perform specific tasks [23].

Health workers were defined as people primarily involved in service provision at primary health care facilities (level 1), their facility-based supervisors (level 2), public health managers whose responsibility was the oversight of primary care facilities (level 3), and national managers who are ultimately involved in strategic decisions regarding services and operations and policies guiding those staff and facilities (level 4).

To generate an initial list of HIS competencies and begin drafting the framework, a systematic literature search was conducted in four major electronic databases using a combination of search terms (Table 1). We used search terms individually and combined in multiple ways using Boolean operators (AND, OR). Database searches were limited to English-language only and covered the period 1990–2012. We applied a snowballing method to the reference lists of all literature to build the bibliography.

**Table 1:** **Literature review search strategy**

**Type**	**Search site**	**Search terms**
Peer-reviewed journal articles (1990–2012)	Science Direct	HIS and competency building
	Pub Med	Health information use and competency building
	Cumulative Index to Nursing and Allied Health Literature (CINAHL)	HIS and competency training
		HIS district-level staff
	Google Scholar	HIS and clinicians, nurses
		HRH and HIS
Government reports	Internet search engine (google.com)	HIS standards
		HIS policy

**Figure 1:**
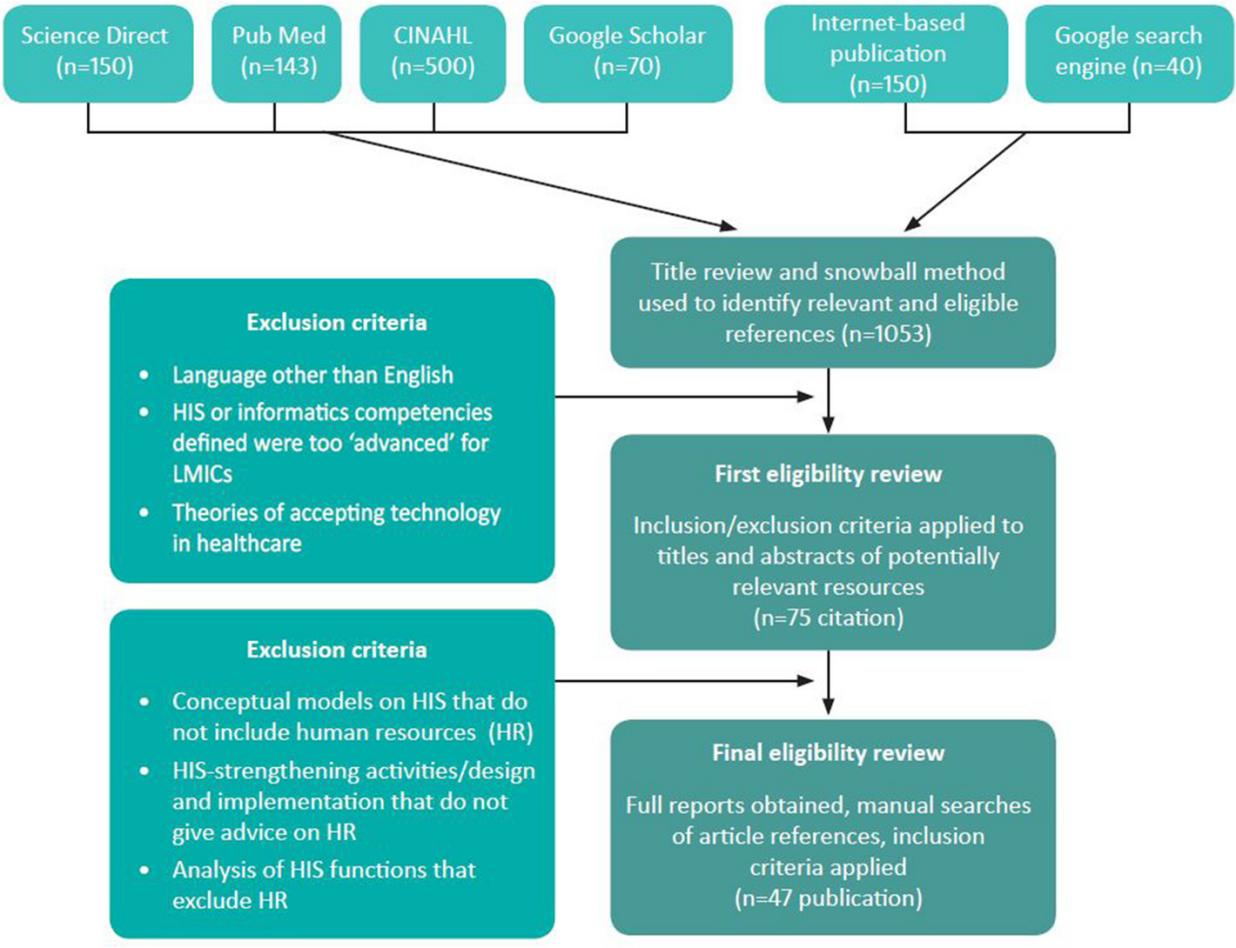
**Flowchart of literature review and selection of articles for analysis.**

The initial search yielded 1 053 publications and resources, which were subject to inclusion/exclusion criteria as demonstrated in Figure 1. From the 47 publications and resources included in the final literature review, we identified the definitions, roles, and competencies that health workers require to undertake routine HIS activities in their level of the health system. We synthesised multiple competencies from the review and compiled them to produce the draft competencies framework for use in expert consultations.

We then created a quantitative survey instrument based on the Delphi method [31]. Examples of the questions for each round appear in Additional file 1. Similar formats were used for each level defined. The Delphi method is a widely used and accepted method for gathering data from respondents within their domain of expertise. It is a group communication process that uses a series of questionnaires delivered in multiple iterations to collect data from a panel of selected subjects, to arrive at a convergence of opinion. The literature review extracted several studies that had used the Delphi method to investigate competencies for health professionals [8,28,29]. We felt this approach was pertinent to our activity as it provided a structured process, but one that also allows qualitative information to be captured.

We purposively sampled 38 experts with broad-based HIS knowledge and extensive experience working in development settings, especially at the field and operational level in Asia, Africa, and the Pacific. Experts were identified from their attendance at the 2011 Asia Pacific Leadership Forum on Health Information Systems in Manila and from the Health Information Systems Knowledge Hub (University of Queensland) mailing list. Letters were sent inviting the experts to participate over the full two-round process over an 8-week period (4 October–23 November 2012). Those who agreed to participate signed informed consent forms and became the expert panel for the consensus-building process.

### First-round consultation

The objective of the first round of consultation was to establish the validity of the “concept”. That is, concurrence that there are general and specialised HIS competencies required at various levels of the health system and that these could be developed into a framework to inform HRH development. In response to the literature review, and building on the method used in previous research [29], we defined four levels of practice for health staff in LMICs:

1. Service providers
2. Facility-based supervisors
3. Provincial-/district-level managers
4. National strategic decision-makers.

In the first round, conducted over a 2-week period (with reminders sent allowing a 2-week extension), we asked respondents if they agreed or disagreed with the classification of the above levels of practice and whether they thought there were general and specialised HIS competencies for each level. An open discussion of the reasons for their responses, further comments, and additional insights were captured. Responses from the first round were analysed and used to modify the draft competencies framework and the survey tool in preparation for round two.

### Second-round consultation

In the second round of consultation, we presented the initial sample of 38 experts with a draft competency framework using the four practice levels and list of HIS competencies derived from the literature. We provided an additional list of competencies for generic information and communication technology (ICT) skills for comment. We asked respondents to move competencies between levels and to add or delete competencies as they saw fit.

The framework listed over 70 competencies mapped against the four levels of practice. A number of general health information competencies were listed in the lower service levels and built upon with specialisation and progression up the levels. We presented this framework to the expert panel using a similar question format to the first round with the aim of determining whether there was agreement with the content, reasons for any divergence or disagreement, whether additional strategic information categories were required, and to allow further comment or clarification on these categories. Respondents were provided 3 weeks to complete this survey, with a follow-up letter providing a 2-week extension. Responses from the first- and second-round surveys were analysed using the Qualtrics® software, and content analysis was performed on open-ended answers. The final framework was peer reviewed by health system and HIS experts, as well as shared with all participants for comments.

## Results

In round one, 17 experts agreed to participate in the consultation and 11 (65%) completed the survey. Responses were received from regions of Southeast Asia, the Western Pacific, Europe, and the Americas (Table 2). In the second round of consultation, 11 experts agreed to participate and eight (73%) completed the survey. Key themes emerging from the feedback are discussed below.

**Table 2:** **Respondent characteristics**

**Characteristic**		**Total sample**	**Round one respondents**	**Round two respondents**
Gender	Male	21	7	7
	Female	17	4	1
Usual place of residence	Southeast Asia	13	5	3
	Western Pacific	6	2	2
	Africa	0	0	0
	Europe	4	1	1
	Americas	15	3	2
	Eastern Mediterranean	0	0	0
Currently working in a HIS role in a LMIC	Yes	—	6	—
	No	—	4	—
	Not stated	—	1	—
Profession	HIS architecture, development, and implementation	—	3	—
	HIS analysis, information production, and dissemination	—	2	—
	Other	—	3	—
	Not stated	—	3	—

### Developing a minimum HIS competencies framework

Respondents agreed there was a need for all health workers to have some basic HIS competencies and that there was validity to the concept of a framework (64% agreement rate). One respondent expressed concern that putting competencies against the four established levels would form a rigid hierarchy and suggested that it would be better to identify general tasks that all positions across the health system should perform. For example, all health workers collect data in some form and it is really the scope and scale of such activities that differs at various levels of the system.

**Figure 2:**
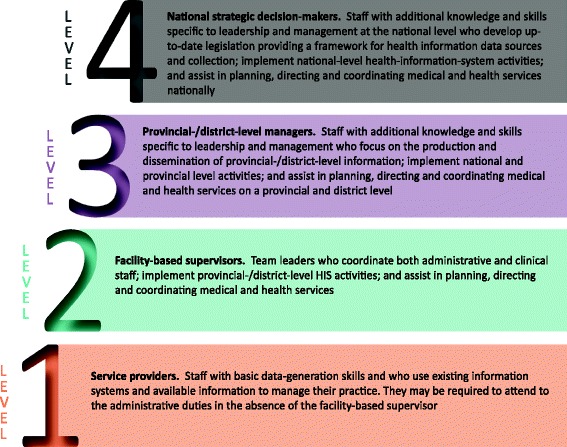
**Roles and responsibilities related to health information at different levels of a health system.**

Several experts commented that these levels should be viewed somewhat arbitrarily as they are not always clearly delineated, and responsibilities at any level are dependent on factors such as the level of decentralisation in the health system and the levels of staffing at particular facilities. For example, in small facilities with only one service provider, that staff member may need to take on duties that span both levels one and two. Finally, there was consensus that competencies around ICT were not relevant for all LMIC settings and therefore should not be included as core competencies. Respondents provided feedback as to how the roles at each level should be elaborated, resulting in the descriptions as demonstrated in Figure 2.

### Core HIS competencies

Consensus (defined as 50% or more agreement rate) was reached around the inclusion of 68 competencies stretching across four levels of the health system (Table 3). The competencies in level one are viewed as core competencies that are built upon and each level adds competencies on top of the previous. Regarding competencies around ICT, the selected competencies were thought to be relevant across all levels that had necessary ICT infrastructure (i.e. computers, internet, and software).

**Table 3:** **Health information competencies by health system level**

**Level 1: facility-based service providers**	**Level 2: facility-based supervisors**	**Level 3: provincial-/district-level managers**	**Level 4: national strategic decision-makers**
*Data management (including use of administrative processes and systems), oversight, and coordination*			
• Uses administrative processes for longitudinal patient monitoring and practice management (e.g. searches for patient records, retrieves demographics)	• Uses administrative processes for maintaining employee records	• Manages projects and provincial-/district-level administration	• Plans and coordinates the national-level surveillance and response activities across all levels of the health system during public health emergencies
	• Uses administrative processes for budgeting	• Develops HIS capacity-building activities across the health system	• Develops up-to-date legislation and health information policies and procedures that provide the framework for implementing the national HIS standards
	• Uses administrative processes for staff scheduling	• Understands provincial/district minimum core health indicators that have been identified for the country (health status and determinants; inputs, outputs, and outcomes of the health system)	• Oversees and monitors the operations/functions of the HIS at the Ministry of Health
	• Uses administrative processes for forecasting facility-level resource allocation	• Establishes coordination mechanisms for the provincial/district statistics office	• Develops up-to-date legislation and health information policies and procedures that provide the framework for implementing the national HIS standards
	• Organises staff workshops and training sessions related to HIS strengthening		• Oversees and monitors the operations/functions of the HIS at the Ministry of Health
	• Uses a systematic approach to evaluating the quality of services provided by health facilities according to national HIS standards		
*Data generation (collection, analysis, management, and dissemination)*			
• Documents patient care using appropriate forms (in accordance with national and/or facility standards)	• Reports regularly on facility supplies, infrastructure, human resources, commodities, budget, and equipment	• Reports regularly on a minimum set of core indicators	• Disseminates health reports and national HIS standards to lower level health facilities and offices
• Enters patient data and facility-level health indicators using appropriate forms (in accordance with the national HIS standards)	• Uses facility and national HIS standards for proper filing and storage of confidential data	• Develops reports for the provincial/district Health Account	
• Accesses and retrieves data at facility level for patient care and health service administration (e.g. filing system)	• Compiles and manages aggregate data	• Follows national HIS standards for data management, analysis, and use at provincial/district level	
• Completes and submits all forms (i.e. weekly/monthly summary and surveillance reports) to the district level health office in a timely manner using the correct practice for paper-based documentation (according to national and/or facility standards)	• Accesses shared data sets	• Ensures accuracy of data collection at health facility	
• Undertakes proper coding/classification, filing, and storage of confidential data as per national HIS data entry and facility standards and as appropriate at the facility level (e.g. diagnosis)	• Uses and understands diagnostic coding	• Publishes most recent summary reports for local authorities	
• Performs transcription, analysis, and compilation of data as required by district and/or national health office		• Disseminates health information used by provincial/district and facility-level management teams to set resource allocation in the annual budget	
• Maintains privacy and security of confidential data		• Provides information and analysis to health facilities and other administrative units as per national HIS standards	
• Creates documentation that is thorough and legible		• Develops integrated HIS summary reports including minimum set of core indicators as per national HIS standards	
		• Accesses, processes, and analyses facility level data as per national HIS standards	
		• Extracts data from clinical and public health data sets	
*Data use*			
• Uses or provides surveillance data to respond to outbreaks according to national standards	• Uses patient records and/or facility health reports to monitor health outcomes	• Uses health information in developing the annual budget plan	• Uses health information in national strategic planning and budget development
• Uses or provides information on forms (national and/or facility standards) to plan patient care (e.g. discharge planning)	• Uses facility-level reports for decision-making on facility-level resource allocation (e.g. supplies, human resources, finances)	• Uses health information at all levels for managing delivery of the local health service and for continuous monitoring and periodic surveillance and response	• Uses integrated HIS summary reports including a minimum set of core indicators to set up strategic management agendas
• Uses or provides facility-retained patient medical records to support quality and continuity of care	• Uses data to evaluate service delivery and health outcomes and to improve quality of care at the facility level		• Uses lower level reports and national HIS reports for future HIS directional planning
• Uses or provides data relating to practice and care as per national HIS standards			• Uses health information in annual national planning and budget development
*Use of ICT infrastructure (subject to availability and applicability at each level)*			
• Uses available communication infrastructure (e.g. fax, telephone, computer, copier, internet, email)		• Routinely saves and backs up files	
• Demonstrates basic technology skills (e.g. able to operate computer, print documents, load paper, change toner)		• Operates virus-scanning processes	
• Demonstrates keyboard skills (i.e. typing)		• Uses presentation graphics (e.g. Excel graphs/PowerPoint)	
• Uses operating system applicable to role (e.g. copy, paste, delete, manage files, change directories, adjust monitor and settings)		• Uses applications for structured data entry (e.g. patient data, service data)	
• Uses computer technology safely and securely		• Uses administrative applications to collate data and develop reports at facility level for decision-making (e.g. customised databases or HIS software applications)	
• Operates peripheral devices (e.g. handheld, scanner, portable storage devices)		• Uses networks to navigate systems (e.g. local area networks, world wide web)	
• Uses surge protection if provided		• Undertakes simple preventive maintenance of computer (e.g. operating system and software updates)	
• Uses word processing (e.g. Microsoft Word)		• Uses aids for clinical decision-making or service decision-support systems	
• Uses spreadsheets (e.g. Microsoft Excel)			

#### Service-provider-level competencies

HIS competencies at this level of the health system focus on the collection and compilation of data from routine clinical forms. Additionally, workers are expected to be able to interpret data for use in clinical practice. Experts agreed that staff at this level should be able to access, document, and analyse data to ensure that a high quality of care is provided to patients. Following standard procedures, such as ensuring privacy of data and legibility of written reports, were also seen as core competencies.

#### Facility-based supervisor competencies

As well as data collection, results showed that facility-based supervisors should have competency in checking the quality of data and interpreting results for use in facility management. Specific competencies include using data for budgeting, planning and scheduling, monitoring health outcomes, and improving the quality of care provided at the facility level. These skills include the ability to “crunch numbers”, disseminate information, and provide feedback to both higher levels and to facility-level staff [32,33].

#### District-/provincial-level competencies

Staff at the district and/or provincial level were described as needing skills in collecting data and checking quality, reviewing and disseminating summaries, and forwarding data to appropriate departments and agencies. Core competencies included the following: the appropriate use of core indicator sets, encouraging coordination between agencies, ensuring the accuracy of facility data, developing integrated HIS reports, and using data for monitoring and evaluation.

#### Regional-/national-level competencies

At the upper levels of the health system, staff should have competency in creating data summaries, analysing data, disseminating findings, and using data for planning. Other key competencies include using data to respond to emergencies and for overseeing health system operations and functions. Using data for resource allocation, HRH planning and development, purchasing, and distribution were also listed.

Excluding the 17 ICT competencies that are considered relevant at all levels, the breakdown of the remaining 51 competencies per level and competency area is shown in Table 4. This analysis reveals that almost half (*n* = 24) of the competencies across the four levels are associated with data generation, particularly at levels one and three. The number of competencies relating to data usage was quite similar across all levels. The management and stewardship competencies demanded more from staff at the higher levels of the system. Table 4 also shows that there are approximately 13, 14, 18, and 8 new competencies for levels one, two, three, and four, respectively.

**Table 4:** **Health information competencies by type and level**

**Competency type**	**Number of competencies**			
	**Level 1**	**Level 2**	**Level 3**	**Level 4**
Data management	1	6	4	3
Data generation	8	5	10	1
Data use	4	3	2	4
*Total*	*13*	*14*	*16*	*8*
*Cumulative total*	*13*	*27*	*43*	*51*

## Discussion

Overall, respondents agreed there was a need for health workers to have minimum HIS competencies as defined in a framework. While there was initial concern over the rigidity of using a framework with defined levels of practice, we incorporated this feedback into the second round of surveys to ensure that it captured generic HIS processes across the system and that complexity and specialisation increased with progression up the hierarchy. We also accepted the four levels of practice as a way of showing the progression of skills and responsibilities expected at higher levels of the system. While there was consensus that ICT competencies were important but not always relevant in all LMIC settings, in order to accommodate countries that have some form of ICT, we decided that ICT competencies should be included as a separate module.

The findings support previous research, which has shown that staff at the facility level should be able to collect, collate and report data, and correctly use standard health system reporting forms [7,32-34]. Competencies relating to data usage were identified at each level, indicating that all staff are expected to not only generate data, but also use data to inform their practice. As the number of new competencies increases at each level, we can infer that tailored training is required for staff to be able to adequately perform HIS tasks at each level of the system.

Several respondents noted that health information is often seen only as the domain of data-entry clerks and HIS specialists such as epidemiologists or information managers. It was evident from the literature that this view of health information as a specialist role has created a widespread attitude among health staff that HIS tasks are additional burdens and not strictly part of their role. This view is confounded by the lack of HIS competencies in training, professional development, and role descriptions. To counteract this finding, health systems need to promote and incentivise a culture of information by ensuring that all personnel are aware of their HIS duties in supporting the health system. Staff also need to recognise that these duties are a core component of *all* roles in the health system.

Although respondents thought the concept of the framework was useful, they stated that staff in LMICs often work across several roles and levels, and thus, the framework would need to be somewhat flexible. Furthermore, many respondents said that although they felt the framework was theoretically sound, in reality, staff in many levels of health systems in LMICs would not have even the basic competencies listed in the first level of the framework and it should thus be applied with careful consideration. The contextual environment of staff present at the facility level, and their minimum competencies, will be important to account for in using this framework to guide capacity-development activities. An important next step in the progression of this framework is to present it with health staff in LMICs, to seek their feedback and recommendations for refinement.

If the framework is to be used to inform training or curriculum development, it may be beneficial to prioritise the competencies or further refine a shortlist of five or six essential competencies for initial focus. Defining competencies is of course only one step in developing a health workforce that is skilled to support health system functioning. Ideally, the ministry of health in each country should take responsibility for creating and enforcing a minimum level of HIS task competence in alignment with the promotion of a culture of information and a supportive environment that provides adequate training and supervision.

In addition to the agreed competencies, there were many that did not reach consensus. This is not surprising considering the diverse professional backgrounds and interest areas of the experts. For example, two sets of competencies created some discussion: the ability to use data presented in geo-referenced formats and ICT competencies such as an understanding of enterprise architecture and system interoperability. While the majority of respondents thought the competencies were too advanced, several felt these competencies should be included at the provincial and national levels. Other research has discussed the need for workers to have presentation skills such as using cumulative graphs and pie charts, and the ability to calculate simple rates and their change over time [35], but these more advanced skills using geo-referenced data have not been noted as core competencies.

A late response from one respondent felt that a key level four data-management competency “defines HIS information and work flows and maintains a set of health data standards to foster systems interoperability” should be included, as should the data-generation competency “develops clear health data and indicator definitions with metadata”. This respondent also pointed out a key omission: in the top three levels of the system, providing feedback on HIS tasks should be included as a key competency. The respondent argued that the flow of information should be bi-directional with reporting being complemented by performance feedback. We received this feedback after the Delphi rounds for arriving at a consensus had been completed, and therefore, these competencies have not been included in the framework, although they warrant serious consideration for future revisions.

Some respondents felt that competencies around generic management skills, such as change management, risk management, and good communication and networking, should be included in the higher level skill sets. However, as we intended to keep the list of competencies focused on those specific to HIS, these sorts of generic skills were not included. Others items that did not reach a consensus for inclusion included competencies around cost-benefit analyses, and competencies focused on surveillance, and monitoring and evaluation. A few respondents wished to have competencies based around specific types of data, for example demographic or statistical data. We did note this type of qualitative feedback, but our ability to delve into specific content areas was outside the scope of the present activity.

A major limitation of the current activity is the restriction to English-only articles. While the majority of publications to-date on this topic have been in English, we note that pertinent information may have been missed in the literature review. Further, while the Delphi method usually involves at least three rounds of surveying, there were only two rounds of consultation carried out due to time constraints. As discussed, this has had a limiting effect on the results, as late responses from round two were not able to be shared in round three. However, these responses have been included in our discussion.

## Conclusion

Based on the initial competencies identified in the literature review and after two rounds of consultation with experts via a Delphi method, 68 HIS competencies (51 core and 17 ICT-specific) were identified in this consultation. The competencies focused on both the generation and use of data at all levels of the health system, highlighting the importance of embedding a culture of information use. This activity is one of the first to try and identify the HIS competencies required among general health workers, as opposed to specialist HIS roles. It is also one of the first attempts to develop a framework on minimum HIS competencies needed in LMICs, which highlights the skills needed at each level of the system, to allow a more systematic approach to HIS capacity building among HRH. Based on information generated from the consultations and in light of current information requirements now mandated by global initiatives, it is strongly recommended that data competency training should be included in professional pre-service and in-service settings.

## Additional file

Additional file 1:
**Survey questionnaires.** This PDF file contains examples of questions used in round one and two of the consultation process. Similar questions were asked for each of the four levels of the health system defined.
