# Supplementary material for: Preparing for the data revolution: identifying minimum health information competencies among the health workforce
Source: Hum Resour Health. 2015 Apr 1;13:17. doi: 10.1186/s12960-015-0002-x (PMC4391110; doi:10.1186/s12960-015-0002-x)
Supplement: Additional file 1: — Survey questionnaires. This PDF file contains examples of questions used in round one and two of the consultation process. Similar questions were asked for each of the four levels of the health system defined. [file 12960_2015_2_MOESM1_ESM.pdf]

Round one survey

Examples of questions used (full survey is available upon request from lead author)

Ref	Question	Options
Q1	Based on our literature search we have identified general HIS roles required for optimal functioning across the health system in developing countries. Do you agree or disagree with the four levels of general HIS roles identified in this list?<br>1. Level 4 - Senior strategic decision-maker<br>2. Level 3 - Provincial/District level managers<br>3. Level 2 - Facility based supervisors/managers<br>4. Level 1 - Service providers	❍ Agree (1)<br>❍ Disagree (2)
Q2	Provide comments as to why you agree/disagree with the four levels of general HIS roles identified, please provide a brief description why	Open text
Q3	Applying the same framework, we now attempt to outline specialized HIS roles across the health system in developing countries. Do you agree or disagree with the levels of specialized HIS roles identified in this list?<br>1. Level 4 - HIS Innovators<br>2. Level 3 - HIS Specialist<br>3. Level 2 - Experienced HIS<br>4. Level 1 - Basic HIS	❍ Agree (1)<br>❍ Disagree (2)
Q4	Provide comments as to why you agree/disagree with the four levels of specialized HIS roles identified, please provide a brief description why	Open text
Q11	These questions describe each of the four levels of general HIS roles in detail. There will be space for you to respond after each role description.<br>Level 1 – Service providers:<br>• Has basic information management and computer technology skills<br>• Use existing information systems and available information to manage their practice	❍ Agree (1)<br>❍ Disagree (2)
Q12	If a characteristic is missing or if you agree/disagree with this list, please list your ideas below and provide a brief explanation	Open text
Q13	If you believe we have missed a key general role (other than the four general roles we have listed), please list this component below and briefly explain your reasons for this inclusion (e.g., you may feel that a general role should be eliminated or added to our list, and provide an explanation based on your opinion)	Open text
Q19	These questions describe each of the specialized HIS roles in detail. There will be space for you to respond after each role description.<br>Level 2 – Basic HIS:<br>• Has fundamental information management and computer skills<br>• Uses existing information systems and available information to manage practice<br>• Record data based on specified indicators for data collection purposes	❍ Agree (1)<br>❍ Disagree (2)
Q20	If a characteristic is missing or if you agree/disagree with this list, please list your ideas below and provide a brief explanation	Open text
Q21	If you believe we have missed a key specialized role (other than the 4 specialized roles we have listed), please list this component below and briefly explain your reasons for this inclusion (e.g., you may feel that a specialized role should be eliminated or added to our list, and provide an explanation based on your opinion)	❍ Agree (1)<br>❍ Disagree (2)

Round two survey

Q1. Please select agree or disagree for each of the general HIS competencies listed below.
Level 1 – Service provider / General HIS competencies:

	Please select	
	Agree (1)	Disagree (2)
1 uses administrative processes for practice management (e.g., searches for patient, retrieves demographics)	❍	❍
2 uses administrative coding/classification as per national HIS data entry standards and as appropriate at the facility level (e.g., diagnosis)	❍	❍
3 uses communication infrastructure available at the facility (e.g., fax, telephone, email)	❍	❍
4 uses data sourcesthat relates to practice and care as per national HIS standards	❍	❍
5 accesses, enters, and retrieves data at facility level for patient care and health service administration (e.g., filing system)	❍	❍
6 uses facility level reports for decision making on facility level resource allocation (e.g., supplies, human resources, finances) at the suitable level within a defined country	❍	❍
7 uses forms (national and/or facility standards) to document patient	❍	❍
8 uses forms (national and/or facility standards) to plan care for patients (e.g. discharge planning) - facility specific	❍	❍
9 uses forms developed at national level to enter patient data and health indicators specific to the level and national HIS standards	❍	❍
10 documentation thorough and legible	❍	❍
11 forms are completed using a timely and proper paper documentation practice (national and/or facility standards)	❍	❍
12 regular reporting on facility budgets	❍	❍
13 regular reporting on facility supplies and commodities	❍	❍
14 regular reporting on facility human resources	❍	❍
15 regular reporting on facility equipment and physical infrastructure	❍	❍
16 uses facility standards for proper coding, filing and storage of confidential data	❍	❍
17 maintains safety and security of confidential data	❍	❍
18 uses patient records and/or facility health reports to monitor facility health outcomes	❍	❍
19 performs transcription, analysis and compilation of data files (data sources) when required by district and/or national level health office	❍	❍
20 submits weekly or monthly surveillance report on time to district level health office (or as per national HIS standards)	❍	❍
21 use of facility-retained patient medical records to support quality and continuity of care	❍	❍
22 uses surveillance data to respond to outbreaks according to national standards	❍	❍

Ref	Question	Options
Q2	Could you suggest any other general HIS competencies for Level 1, that are not defined in Question 1	Open text
Q3	Please provide any additional comments about general HIS competencies for Level 1 here	Open text
Q4	In health facilities where the supervisor role is vacant, the service provider performs both supervisory and clinical practitioner roles. Therefore, Level 2 (Facility based supervisors) have all the competencies listed in Level 1 (Service providers), including the additional competencies for Level 2	❍ Agree (1)<br>❍ Disagree (2)

Q15. Please select agree or disagree for each of the ICT skills as part of HIS competencies listed below for all four levels of HIS (if computers are available).

	Please select	
	Agree (1)	Disagree (2)
61 demonstrates basic technology skills (e.g., load paper, change toner, print document)	❍	❍
62 demonstrates keyboard (i.e., typing) skills	❍	❍
63 simple user based preventative maintenance of computer	❍	❍
64 save and backup files	❍	❍
65uses operating system applicable to role (e.g., copy, paste, delete, change directories)	❍	❍
66 uses computer technology safely	❍	❍
67 operates peripheral devices (e.g., hand held)	❍	❍
68 operates virus scanning process installed	❍	❍
69 uses surge protection if provided	❍	❍
70 uses word processing	❍	❍
71 uses spreadsheet	❍	❍
72 (If available) uses presentation graphics (e.g., Excel graphs /Power Point)	❍	❍
73 uses administrative applications for practice management (e.g., searches for patient, retrieves demographics	❍	❍
74 uses applications for structured data entry (e.g., patient data, service data)	❍	❍
75 uses aids for clinical decision making or service decisions support systems	❍	❍
76 uses administrative application to collate data and develop reports at facility level for decision-making purposes	❍	❍
77 uses networks to navigate systems (e.g., world wide web) when applicable or available	❍	❍
